# Improving Simulation Model Accuracy for Friction Stir Welding of AA 2219

**DOI:** 10.3390/ma18051046

**Published:** 2025-02-27

**Authors:** Kennen Brooks, Bryan Ramos, David J. Prymak, Tracy W. Nelson, Michael P. Miles

**Affiliations:** Department of Manufacturing Engineering, Brigham Young University, Provo, UT 84602, USA; kennen_brooks@steris.com (K.B.); bryan.ramos.gonzales@cummins.com (B.R.); john@prymakprecision.com (D.J.P.); tracy_nelson@byu.edu (T.W.N.)

**Keywords:** friction stir welding, numerical modeling, simulation, aluminum alloys, friction laws, material flow stress

## Abstract

Modeling of friction stir welding (FSW) is challenging, as there are large gradients in both strain rate and temperature (typically between 450 and 500 °C in aluminum alloys) that must be accounted for in the constitutive law of the material being joined. Constitutive laws are most often calibrated using flow stresses from hot compression or hot torsion testing, where strain rates are much lower than those seen in the stir zone of the FSW process. As such, the current work employed a recently developed method to measure flow stresses at high strain rates and temperatures in AA 2219-T67, and these data were used in the development of a finite element (FE) simulation of FSW. Because heat generation during FSW is primarily a function of friction between the rapidly spinning tool and the plate, the choice of friction law and associated parameters were also studied with respect to FE model predictions. It was found that the Norton viscoplastic friction law provided the most accurate modeling results, for both the transient and steady-state phases of an FSW plunge experiment. It is likely that the superior performance of the Norton law was its ability to account for temperature and rate sensitivity of the plate material sheared by the tool, while the Tresca-limited Coulomb law favored contact pressure, with essentially no temperature or rate dependence of the local material properties. With optimized friction parameters and more accurate flow stresses for the weld zone, as measured by a high-pressure shear test, a 65% overall reduction in model error was achieved, compared to a model that employed a material law calibrated with hot compression or hot torsion test results. Model error was calculated as an equally weighted comparison of temperatures, torques, and forces with experimentally measured values.

## 1. Introduction

Friction stir welding (FSW) is a solid-state joining process that was invented by The Welding Institute, Cambridge, U.K., in 1991 [1]. FSW methods have been developed for many different alloys and weld geometries, but this often requires significant trial and error experimentation. The process parameters and tool design needed to achieve a good weld can be different for each material and weld geometry, hence the need for time-consuming trial and error [1,2]. Accurate numerical simulation of FSW could help to reduce the time and effort needed for welding development, but after many years of effort, the predictions generated by FSW models have often not proven accurate enough to be useful.

Most prior friction stir welding (FSW) modeling efforts employ data from hot compression or hot torsion testing to inform material constitutive behavior, because the flow stresses generated by these methods are readily available [3,4,5]. These standard tests simulate more standard deformation mechanisms [6,7,8] rather than the high-shear conditions that occur next to a rapidly spinning FSW tool. Rather than questioning whether these data are appropriate for simulating FSW, most investigators tune the model by adjusting the friction coefficient and/or heat transfer coefficients to achieve reasonable temperature predictions. Recent work in AA 6061-T6 has shown that a high-pressure shear test provides more realistic flow stresses for modeling a process like FSW [9], because the highly sheared material near the rotating tool is not well replicated in testing methods like hot compression or hot torsion experiments. The purpose of the current work is to determine the effects of friction law parameters and material flow stress data on FSW model results to improve model accuracy.

In FSW, the main process parameters are welding traverse speed, tool rotational speed, tool tilt angle, tool design, axial pressure of the tool on the plate, and tool penetration into the plate. The primary results from a process model are material flow, temperature fields in the tool and plate, stresses, and strains [10]. Heat generation in the FSW process is produced by friction and plastic deformation at the tool/plate interface. Much of the heat is generated near the shoulder, with the highest temperatures in the material at the surface (in contact with the tool) and decreasing through the thickness of the plate [11]. The temperature history experienced by the plate largely determines the properties of the weld while also affecting the residual stresses and distortion that occur after welding [12]. If there is insufficient heat during processing, defects such as poor material flow and poor mixing can form, resulting in defective welds [13]. Due to the large amount of plastic deformation present, FSW exhibits non-Newtonian viscosity for metal flow that requires strain rate- and temperature-dependent flow stresses [14].

The goal of numerical modeling of FSW has ultimately been focused on obtaining accurate predictions that would allow for optimizing the process parameters in order to achieve good weld properties [10], including targeting desirable microstructures in the processed materials [15]. With some limited success, models have even been used to explore and attempt to optimize tool design [10,16]. FSW combines many interdependent physical phenomena, both thermal and mechanical, into one process, making it difficult to model [17]. However, the current models do not adequately address each of these facets, so the prediction cannot be used to optimize the process [10].

In review of prior research done on numerical models of FSW, most models validate either the tool temperatures or the plate temperatures but not both sides of the frictional interface at the same time. This is important, because a model can be tuned by adjusting the friction coefficient to achieve good agreement with measurements in the tool or measurements in the plate, but if the model is not able to predict temperatures in both simultaneously, it is not certain that the heat generation level at the interface is correct or that the partitioning of heat generated by friction is done properly between tool and plate. As such, partially validated models may have some value in providing qualitative trends for an FSW process, but they are not rigorously predictive at a level where welding development could be performed numerically.

There have been many methods used to model friction stir welding. Some of these include two- and three-dimensional versions of thermomechanical models [10,18], weld material models [18], computational fluid dynamics (CFD) models [10,19], non-thermo-mechanical approaches [18], and others [10,18]. In creating a numerical model for FSW, boundary conditions are a key input for both the thermal and mechanical problems. With the thermal side of a model, heat loss through conduction between tool and workpiece, as well as heat exchange with the surrounding environment, are needed to properly predict temperature evolution. Heat is generated by friction and plastic deformation, which is conducted away from the tool/plate interface through the air or other fixtures in contact with the tool or plate, so proper physical data, as well as heat transfer coefficients, are needed to predict temperatures measured during experimental benchmark tests [10]. In addition to the heat generated by friction, there is also a certain amount of heat-generated plastic deformation, so both mechanical processes must be accounted for and properly modeled [20]. As such, the frictional interface between the tool and the workpiece is of critical importance [21]. It was found that most prior work employed either the Coulomb or Norton (viscoplastic) friction laws to model FSW [14,20,22], but when a modeling approach is primarily thermal, as with some CFD models, the heat generated can be simplified into a power input from an analytical expression of torque and rotational speed, then used to validate the overall heat input predicted by experiments while investigating the flow of heat during a weld traverse [23]. A specific application of the numerical modeling of FSW is predicting failures at the weld interface, including various types of void defects [24,25,26]. Residual stresses from FSW have been predicted using a mechanical model in tandem with a thermal model [27]. However, discrepancies were found in the simulated stress results, where the predicted values were within acceptable orders of magnitude but still relatively inaccurate. This highlighted the need for more work in improving numerical models of FSW so they can be used to predict weld performance.

In research focused on numerical modeling for the skew rolling process, for example, different friction laws have been explored to see how they work in a simulation context [28]. Skew rolling is similar to FSW in that it is governed by high shearing of the plate. It was found that the best friction laws for skew roll modeling were Tresca or viscoplastic friction. This was further supported, as other research also employed Tresca [29,30] and viscoplastic friction [14,17]. As such, these friction laws were considered for the current model.

The Tresca friction law models the interface by limiting the resistance of the rotating tool on the plate to the yield stress, in pure shear, of the plate material, given by

τ = *m*k (1)

where *m* is a friction factor, with values between 0 and 1, indicating the amount of relative sliding at the interface, and *k* is the yield stress of the plate in pure shear, defined by

(2) $$k=\frac{\sigma_{eq}}{\sqrt{3}}$$

where $\sigma_{eq}$ is the von Mises equivalent stress [28]. In some cases, the Tresca law is used to provide a limiting saturation to the Coulomb friction law, given by

(3) $$\tau =\left\{\begin{array}{c} \mu p,\;\;\mu p<mk\\ mk,\;\;\mu p\;\geq mk \end{array}\right.$$

where μ is the Coulomb friction coefficient, and p is the normal pressure [29]. This friction model would seem to be appropriate for FSW, as the sliding phase of welding, which is a transient phase, can be modeled by Coulomb’s law, transitioning to the Tresca law after significant heating of the material occurs as the plate is sheared by the rapidly rotating tool.

The viscoplastic friction law, also known as Norton’s friction law, models the shear stress at the interface as a function of the local plate properties and the relative sliding velocity, given by

(4) $$\tau =-\alpha K{\left|v_{rel}\right|}^{pf-1}v_{rel}$$

where α is the friction coefficient, *K* is the material consistency, $v_{rel}$ is the relative velocity at the interface, and $p_{f}$ is the sensitivity to the sliding velocity, which is equivalent to the strain rate sensitivity index for the plate material [28]. However, *K* is not constant and depends on both the temperature and equivalent strain of the material, given by

(5) $$K=K_{0}{\left(\varepsilon \;®+\varepsilon_{0}\right)}^{n}\exp\left(\frac{\beta }{T}\right)$$

where $K_{0}$, $\varepsilon_{0}$, and β are constants, and *n* is the strain-hardening exponent [28]. The viscoplastic friction law essentially models the interface as a boundary layer of material that behaves in a viscous manner, making the shear stress dependent on the sliding velocity. Since FSW shears the material in contact with and around the tool, this law also appears to be appropriate for the modeling of FSW. A description of each friction law used in this study is shown in Table 1.

## 2. Experimental Methods

The primary focus of this work is to model an FSW plunge and to validate the model with experimental results on AA 2219-T76 plate plunge experiments using ForgeNxt^®^ v3.1 software. In so doing, two key issues were studied with respect to the predictive capability of the model. First, the range of parameters that can be adjusted within the friction laws, presented in the last section, were studied in order to understand their effect on the process results and temperature evolution within the plate in order to determine an optimal parameter set. A physical interpretation of these laws with respect to the adjustable parameters was also sought. Second, a new approach to experimentally measuring material flow stresses at high strain rates and temperatures, typical of FSW, has been developed in prior work [9], and data from this new approach were generated and implemented in the model to determine the potential influence in improving the model predictions with respect to the experiment.

In a prior work [9], a set of FSW plunge experiments used a pinless flat tool with varying plunge forces, RPM, and plunge depths to capture a range of data across different parameters. The experimental data used in the current work followed the same approach. The plunge experiments were conducted on AA 2219-T76 plates in a similar fashion but with a hollow tool in order to reduce the dead zone observed in the plate when flat tool plunges were conducted. These experiments served as the benchmark and validation tool for the numerical model.

**Figure 1:**
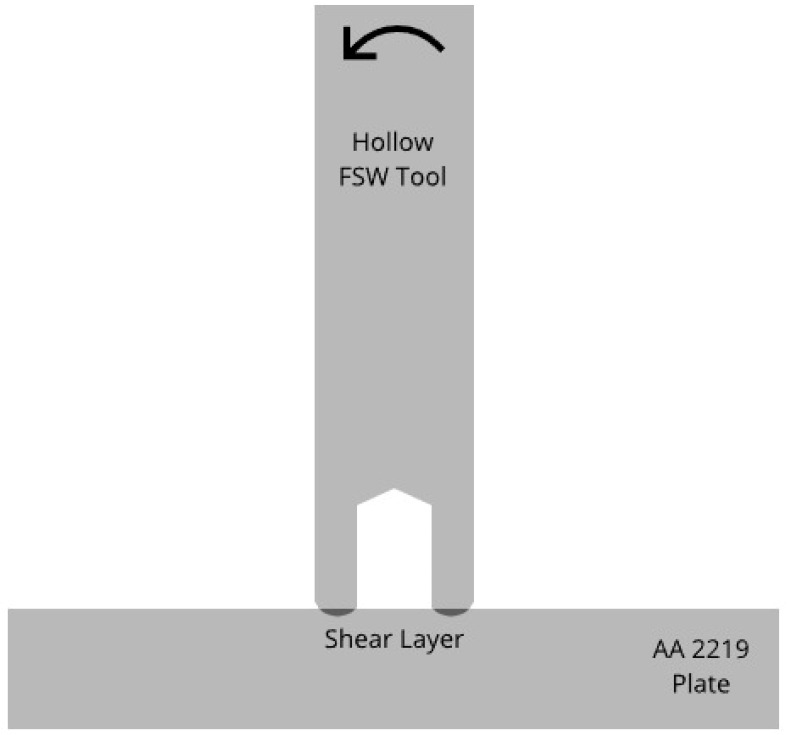
Geometry used for the plunge experiments, where the shaded area under the tool represents the shear layer created by the spinning tool.

**Figure 2:**
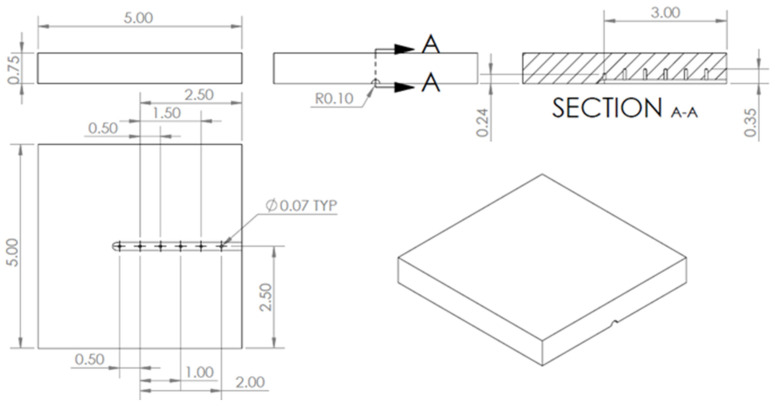
Dimensions and thermocouple locations (in inches) for the AA 2219-T76 plate used in the benchmark experiments.

**Figure 3:**
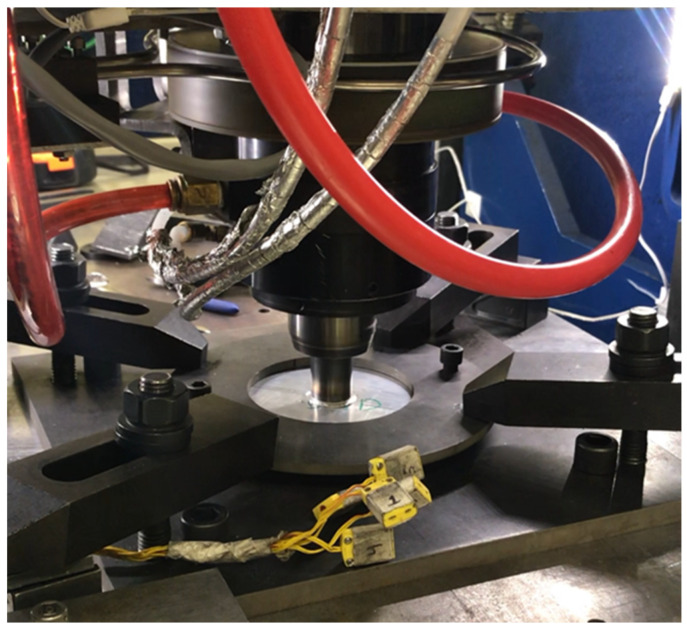
Experimental configuration: friction stir welding machine with plunge tool and plate.

Plunge tests were conducted on AA 2219-T76 plates using a hollow tool made of H13 tool steel (see Figure 1). A hollow tool was used in order to eliminate the dead zone in the plate material that occurs when a solid tool is used. The dimensions and thermocouple locations for the AA 2219 aluminum plate can be seen in Figure 2. The thermocouples were placed well below the stir zone to avoid contact with the tool during processing. The dimensions of the tool were 100 mm in length, an outer diameter of 25.4 mm, and internal diameter of the hollow portion of 13 mm, with a depth of 17 mm. The three thermocouples were placed in the tool spaced 120° from each other at 9 mm, 9 mm, and 13 mm from the center and 2 mm from the tool surface. The plunge experiments were force controlled using a load of 45 kN and a spindle speed of 600 RPM while allowing the tool to plunge to a 2 mm depth before stopping the test. These parameters were chosen from among those provided in a prior study [9], where it was shown that good flow of the material under the tool could be obtained for this set of RPM and vertical load. The time required to reach a depth of 2 mm was relatively quick, at about 8 s, making the corresponding simulation time shorter than some of the other viable parameter sets while still providing adequate processing of the plate. The experimental setup is seen in Figure 3, where a plate specimen is fixtured and the plunge tool has penetrated the top surface of the plate during a plunge experiment.

## 3. Simulation Approach

The vertical displacement data for the tool, measured during the experiments, was used as a boundary condition in the model rather than a constant load boundary condition. This was done to reduce the computation time required to reach a solution at each time increment while achieving the same result, i.e., to impose a constant vertical load on the tool. At each interface in the model, a thermal exchange coefficient was defined, based on values that are typical in the literature [31]. The values used in the current work are shown in Table 2. In addition to the thermal exchange coefficients, the temperature-dependent material properties for both the tool and plate—density, thermal conductivity, and specific heat capacity—were verified to ensure that the heat partitioning at the tool/plate interface would be accurate.

**Figure 4:**
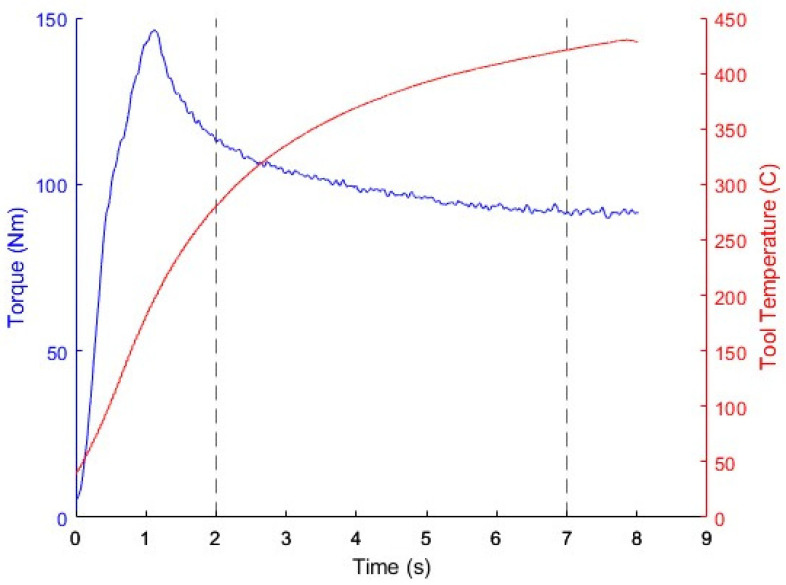
Spindle torque and tool temperature for an FSW plunge. Comparison of the simulation and experimental results were performed at 2 s (“transient” phase) and at 7 s (quasi-“steady-state” phase).

The friction law used at the interface between the rotating tool and the plate was an area of particular interest. It was decided that the Tresca-limited Coulomb and viscoplastic friction laws would be investigated, based on the initial simulation trials and also based on prior work where these laws were employed for the modeling of FSW. The parameters of each friction law were varied and studied to characterize their effect on the plunge simulation results. The results from the various simulations were compared during the transient and quasi-steady periods of the plunge experiments, where torque from the spindle and temperature of the tool were plotted against time, as seen in a typical plot shown in Figure 4. The “transient” period was taken at 2 s, while the “steady-state” period was taken at 7 s.

**Figure 5:**
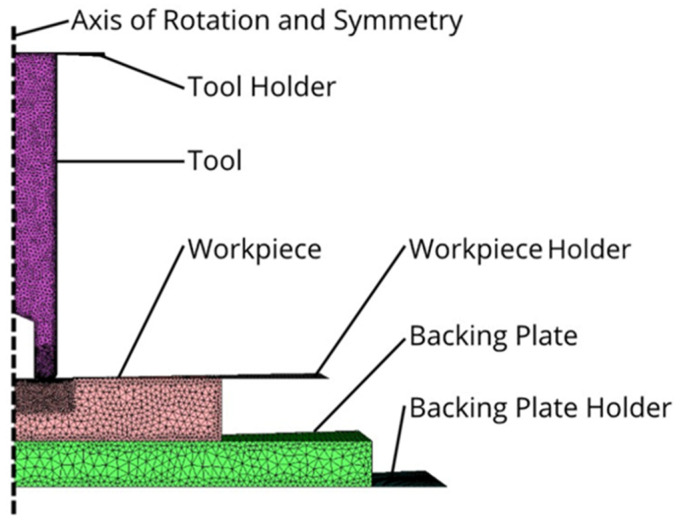
View of a 2D model using a hollow tool in Forge^®^ software, where the axis of rotation is the left edge.

As the modeling was developed using a two-dimensional axisymmetric approach, a plane of symmetry was defined, representing one-half of a section view, as shown in Figure 5. This approach provides an efficient computation, relative to a full 3D model, and can also provide accurate predictions of experiments, as will be seen in the Results section. The 2D axisymmetric approximation does not model local shearing of the material at the weld interface, as the rotational velocity component is not present in this model, but a “virtual” rotational velocity is calculated on the tool surface from its RPM at each radial position on the tool, so that relative sliding velocity with the plate can be calculated for the frictional heat generation.

Heat generated by plastic deformation of the plate is calculated in the classical way as

(6) $${\dot{q}}_{v}=f\bar{\sigma }\dot{\bar{\varepsilon }}$$

where ${\dot{q}}_{v}$ is the volumetric heat generation, $\bar{\sigma }=\sqrt{\frac{3}{2}s:s}$ is the equivalent stress, and the factor f takes into account the fraction of energy converted into heat, taken as 0.9 in this paper. Heat generation from friction at the tool/sheet interface is given by

(7) $${\dot{q}}_{f}=\tau \cdot ∆v_{s}$$

where τ is the friction shear stress given by Equation (3) or Equation (4), and $∆v_{s}$ is the relative sliding velocity at the tool/plate interface. Frictional heat is shared between the plate and tool as a function of the effusivities of each, where the material with higher effusivity receives a greater portion of the frictional heat. Effusivity is defined as $\sqrt{\rho ck}$, where ρ is the density, c is the heat capacity, and *k* is the conductivity.

**Figure 6:**
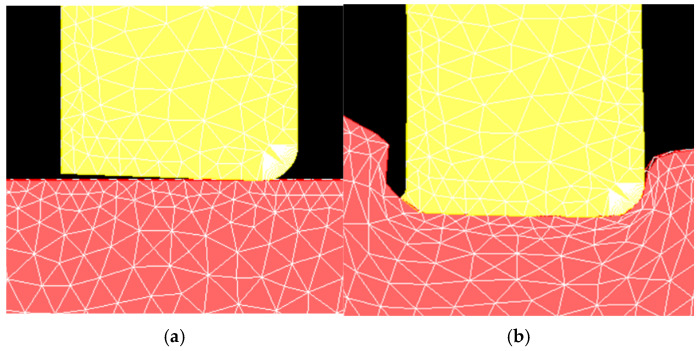
Mesh of the 2D model prior to refinement (**a**) before and (**b**) after the tool plunges. The plastically deformed plate material does not follow the tool surface accurately when the mesh is too coarse.

**Figure 7:**
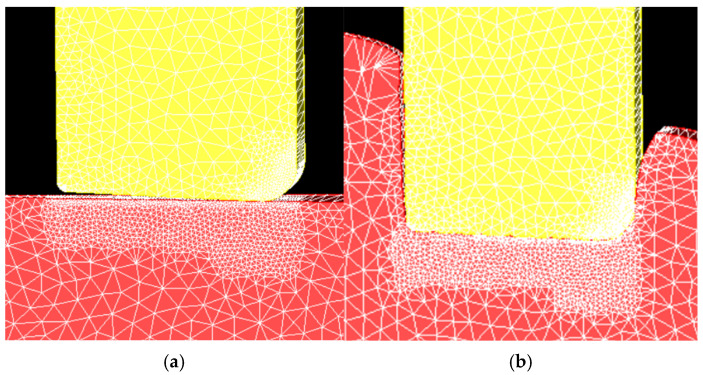
Mesh of the 2D model after being refined (**a**) before and (**b**) after the tool plunges. The plate material better follows the tool surface during the plunge.

**Figure 8:**
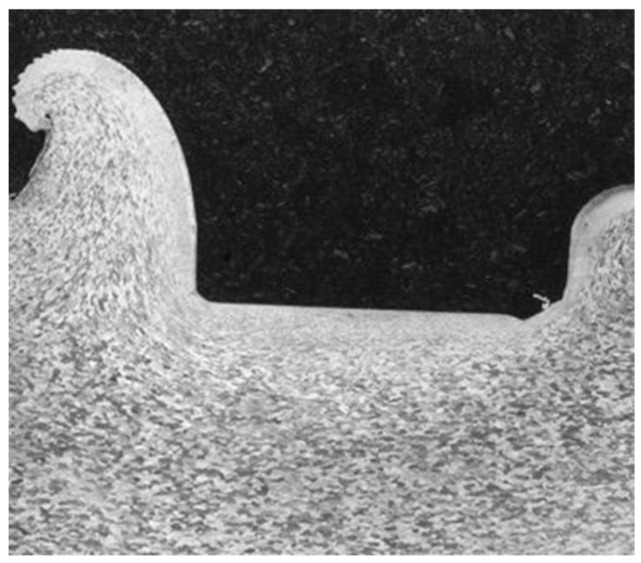
Cross-section of the plate surface after the plunge experiment showing the material flow around the tool.

As part of the initial model development, the mesh needed to be refined to achieve accurate depictions of the material flow behavior. Initially, the plate material separated from the tool in an unrealistic manner. To improve the fidelity of the plastic flow behavior, the plate mesh was refined beneath and near the edges of the tool, with frequent remeshing over the course of the simulation. This allows the nodes on the surface of the tool to better follow its contour while still maintaining the unilateral contact condition, which requires a compressive normal stress at any point if the contact constraints are to be applied by the model. The tool mesh was also refined to provide better resolution of the temperature gradients near its surface. The mesh refinements and their effects can be seen in Figure 6 and Figure 7. While a formal convergence study was not done, the refinement changes were confirmed to render the flow of material realistic with respect to the experimental cross-sections observed after the plunge experiments in this alloy, as seen in Figure 8.

Due to the severe plastic deformation induced in the plate material, especially directly under the tool, it was confirmed in a prior study that a standard set of material flow stress data taken from either hot torsion or hot compression testing may be inappropriate [9]. As such, data from a new high-pressure shear experiment were employed in the zone directly under and around the tool in order to better replicate the experimental conditions in FSW. The stir zone, directly under the tool, was defined with the new, improved flow stress data, while outside of this zone, standard material flow stresses were employed to model the lower deformation areas outside of the so-called stir zone.

The error of the computations, for a series of important variables, was calculated at 0.5 s increments from 0.5 s to 7.5 s, during which the temperatures, loads, and torques were changing as a function of the plunge depth. These half-second buffers were in place to avoid discrepancies in the numerical model that may have occurred when the simulation began or concluded. In computing the error, the simulation results were compared to the experimental data at the same points in time and in the same locations (model and experiment), given by

(8) $$Timestep\;Error=\frac{\sum_{i}^{n}{\left[\frac{\left|Simulated\;Result-Experimental\;Measurement\right|}{Experimental\;Measurement}\right]}_{i}}{Number\;of\;Results}\times 100\%,$$

(9) $$Overall\;Error=\frac{\sum \left[Timestep\;Errors\right]}{Number\;of\;Timesteps}$$

**Figure 9:**
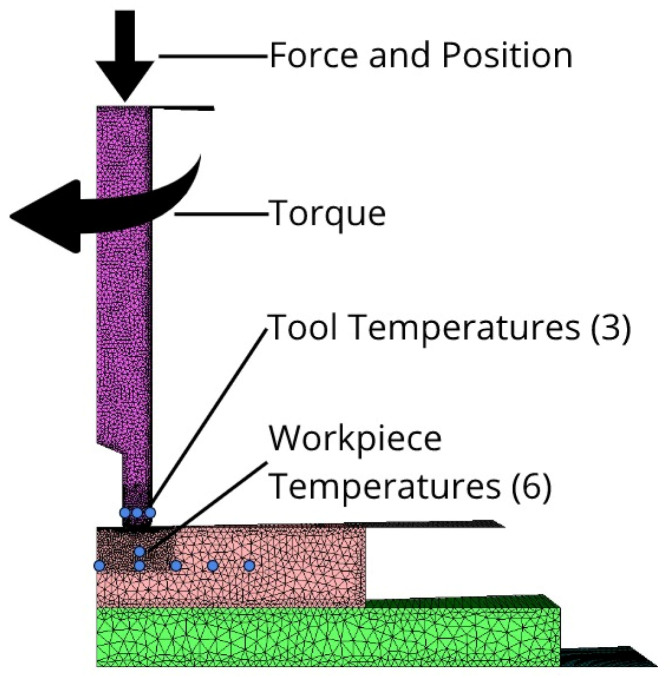
Variables and locations within the model used for error calculation and comparison with the experiment. The blue dots represent the thermocouple locations from the experiment.

In Equation (8), the residuals for each result are normalized using the experimental data, and then, all normalized residuals are averaged together with equal weighting to calculate the error at that timestep. Then, in Equation (7), the errors at each timestep are averaged to determine the overall error. This error scheme provided a way for both relative and absolute comparisons between simulations to quantify the effects of each parameter on the accuracy of the model. The variables used to compute the model error with respect to the experimental measurements are shown in Figure 9 and summarized in Table 3. Note that, because the overall error computed by Equation (7) aggregates the temperature errors from 9 different measurements (6 in the plate and 3 in the tool), in addition to the load and torque errors, the number will appear to be high, but this approach is being used to compare changes to the simulation, so the relative change in error is of interest rather than the absolute value.

## 4. Results and Discussion

### 4.1. Effect of Friction Laws on Model Accuracy

**Figure 10:**
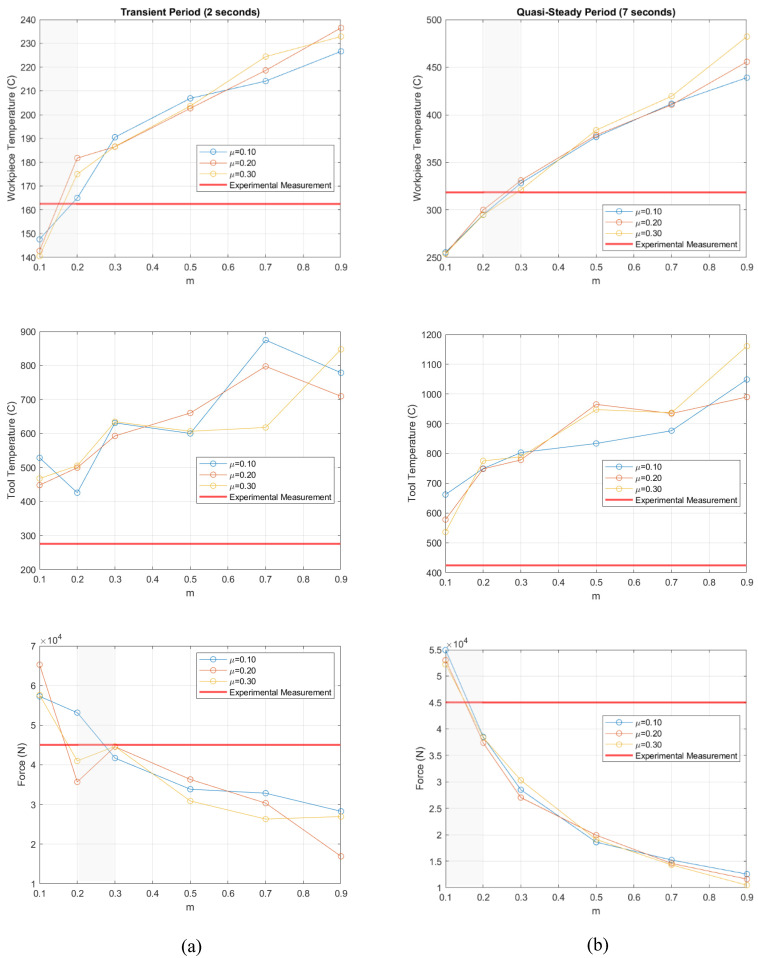
Plate maximum temperature, tool maximum temperature, and vertical tool loads for the Tresca-limited Coulomb friction law during the (**a**) transient and (**b**) quasi-steady periods. The shaded areas indicate the range of the friction factor *m* that is viable in each case.

To characterize the effect of varying friction law parameters, the Tresca-limited Coulomb law was first evaluated, where 18 plunge simulations were performed, varying the *μ* from 0.1 to 0.3 and *m* from 0.1 to 0.9. The results were then compared with the experiment at plunge times of 2 s (transient) and 7 s (quasi-steady-state), as shown in Figure 4. The maximum plate temperature, maximum tool temperature, and vertical tool loads are plotted in Figure 10 as a function of the friction factor *m*. During both the transient and steady-state periods, *m* exhibits a linear relationship with the plate temperatures when greater than 0.30. This linear relationship is not observed for the tool temperature results, but there is still some positive correlation. As for the force results, there was an inverse relationship with *m* that was fairly linear during the transient period but turned quadratic during the quasi-steady period. When looking at the variations in the results due to the changes in *μ*, an increasing sensitivity to *μ* was seen with increasing the *m* for the plate temperatures during the transient and quasi-steady periods due to the limiting Tresca factor being increased. However, overall, the effects of *μ* were not very influential on the results for the range studied.

If the Tresca-limited Coulomb friction law is to be used in an FSW simulation in 2xxx series aluminum, there are general trends and guidelines that can be used to determine the optimal parameter set. First, start with a value of either 0.20 or 0.30 for *m*. Then, focus on increasing or decreasing *m* by 0.10 and then 0.05 to refine the results on a macro scale. If the temperature results are overpredicted or force results are underpredicted, lowering the value of *m* should increase the accuracy of the model. On the contrary, if the temperature results are underpredicted or force results are overpredicted, raising the value of *m* should increase the model accuracy as well and should specifically decrease the force results more than the temperatures increase. Once the results are within a reasonable range of the benchmark or adjustments to *m* no longer provide increased accuracy, adjusting the *μ* value can fine tune the results, starting with a value of 0.20. Similar trends to adjusting *m* were found as stated above, so if the temperatures are underpredicted or force results are overpredicted, increasing *μ* by increments of 0.10 should improve the model accuracy. However, as shown in Figure 10, the effect of *μ* was not as predictable and may need additional study, but following these guidelines should improve parameter selection when using the Tresca-limited Coulomb friction law in an FSW model for 2xxx series aluminum.

The errors for these 18 simulations were calculated across all timesteps to determine the best friction law parameters. The most accurate parameter set used *m* = 0.10 and *μ* = 0.20, resulting in an overall error of 72.7%.

Next, the viscoplastic friction law (see Equation (4)) was explored to determine general trends between the input parameters and simulated results. Eight simulations were run with *α*, the friction coefficient, at 0.30 and 0.40, where changes due to *p*, sensitivity to sliding, were studied by varying it from 0.15 to 0.30. This range was chosen due to 0.15 being the default value within Forge^®^Nxt 4.0 software (Transvalor SA, Sophia-Antipolis, France) that is typical for hot forming [28], while superplastic aluminum can achieve values of 0.5 [32]. Since AA 2219 is not superplastic, a value of 0.30 was used as an upper bound.

**Figure 11:**
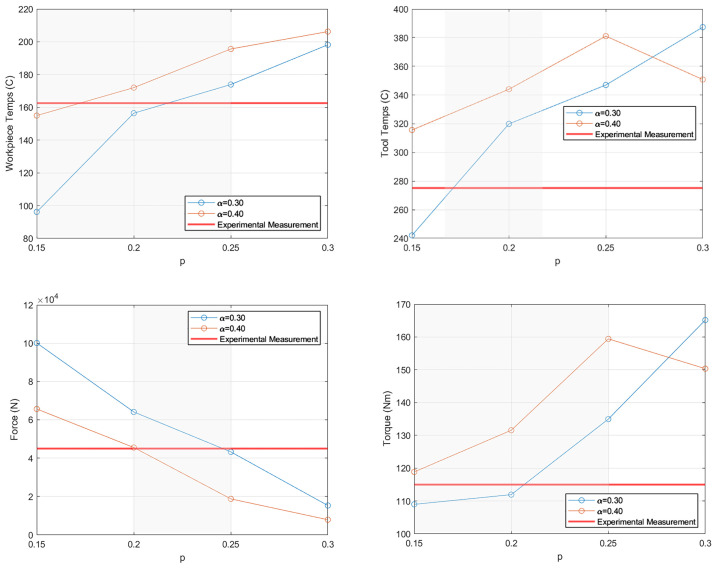
Simulation results from the study of viscoplastic friction during the transient period at 2 s. The shaded areas indicate the range of *p* that is viable for each result.

The simulation results were compared during the transient and quasi-steady-state periods (2 s and 7 s; see Figure 4), as was done for the Tresca-limited Coulomb friction law, but in this case, the torque results were also calculated (see Figure 10 and Figure 11). A general trend at both timesteps shows that, as *α* increased, the temperatures and torques increased while the forces decreased. Changes to *p* showed some non-physical behavior (for *p* = 0.30) for the tool temperatures and torque, as higher friction values resulted in lower temperatures and torques, indicating that a value of less than 0.30 should be used.

Based on these results, if the viscoplastic friction law is chosen for an FSW model, there are guidelines to determining an optimal parameter set. For p, the initial value can be estimated as the strain rate sensitivity of the plate materials. If the plate is aluminum, a value of 0.20 is reasonable. From there, if the temperature or torque results need to be increased, *p* can be increased in increments of 0.05 until a good match occurs, but the *p* value should not be adjusted too much, as it is tied to the material properties. As for adjusting α, there is more flexibility. Beginning with a value of 0.30 as a starting point for α, increase the value by increments of 0.10 to increase the temperature and torque results and decrease the force results, or decrease α if the opposite trend in results is needed. Once the simulation is within the desired accuracy, the parameter set can be used for simulation work.

**Figure 12:**
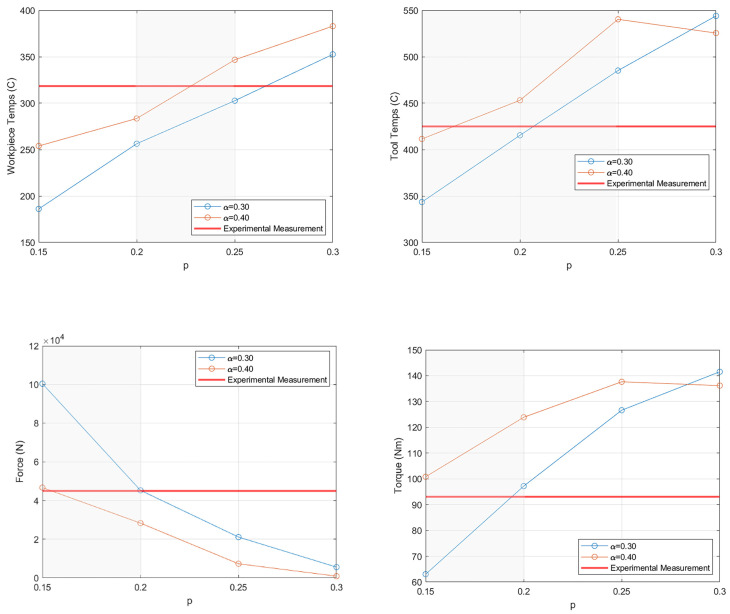
Simulation results from the characterization study of viscoplastic friction during the quasi-steady period at 7 s. The shaded areas indicate the range of *p* that is viable for each result.

From the comparisons of transient and quasi-steady periods, the ideal *p* value fell between 0.15 and 0.25 shown in the shaded areas of Figure 11 and Figure 12. However, the majority of the results had an upper or lower limit of 0.20 for *p*. As there was no clear ideal parameter set, attention was given to a broader look at each simulation instead of only the two snapshots in time (2 s and 7 s). The errors were calculated for each simulation run employing viscoplastic friction to evaluate the effect of the friction parameters used (see Table 4). The results from these errors showed that the best parameter sets were *α* = 0.40, *p* = 0.15 with an error of 53.9%, followed by *α* = 0.30, *p* = 0.20 with an error of 62.3%.

In comparing the simulation results, the lowest error using Tresca-limited Coulomb friction was 72.7% (see above), whereas, using viscoplastic friction, the lowest error was 53.9% (see Table 4). Additionally, when using viscoplastic friction, the model was able to converge better when the torque results were calculated. From this, it was concluded that viscoplastic friction was better suited for an FSW model. The two best viscoplastic friction parameter sets (*α* = 0.40, *p* = 0.15 and *α* = 0.30, *p* = 0.20) were then further studied to determine if the error levels in the predictions could be driven lower by refining the model in areas beyond the friction laws.

In terms of physical interpretation of these friction laws, it was seen that the rate sensitivity of the viscoplastic law, where the parameter *p* essentially represents the rate sensitivity of a boundary layer of plate material being sheared by the tool, was of benefit in reaching more accurate predictions with respect to the experiment. The Tresca-limited Coulomb law, while incorporating the local yield stress of the plate material in pure shear in the calculation of the frictional resistance to the tool rotation, does not account for the rate sensitivity and is therefore less able to adapt to various levels of relative sliding velocity (or, in this case, local shearing of the material) between plate and tool surface.

While the information gained by characterizing the two friction laws increased the understanding of how each parameter affects the accuracy of the model, it showed that the accuracy issues present in the model could not be fixed by only changing the friction parameters. Modifications needed to be made to the other parameters, such as the material properties and other boundary conditions, in order to achieve more accurate model predictions.

### 4.2. Effect of Local Flow Stresses on Simulation Accuracy

**Figure 13:**
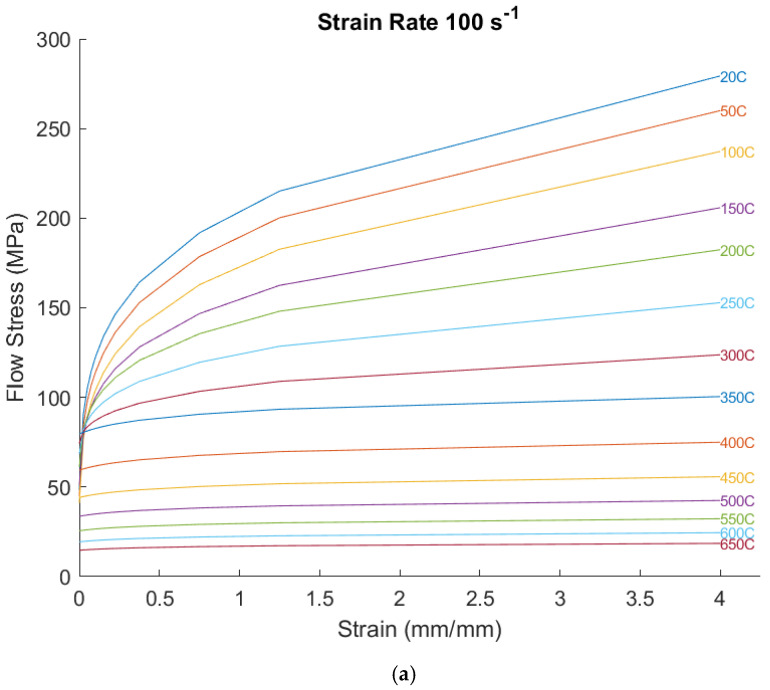

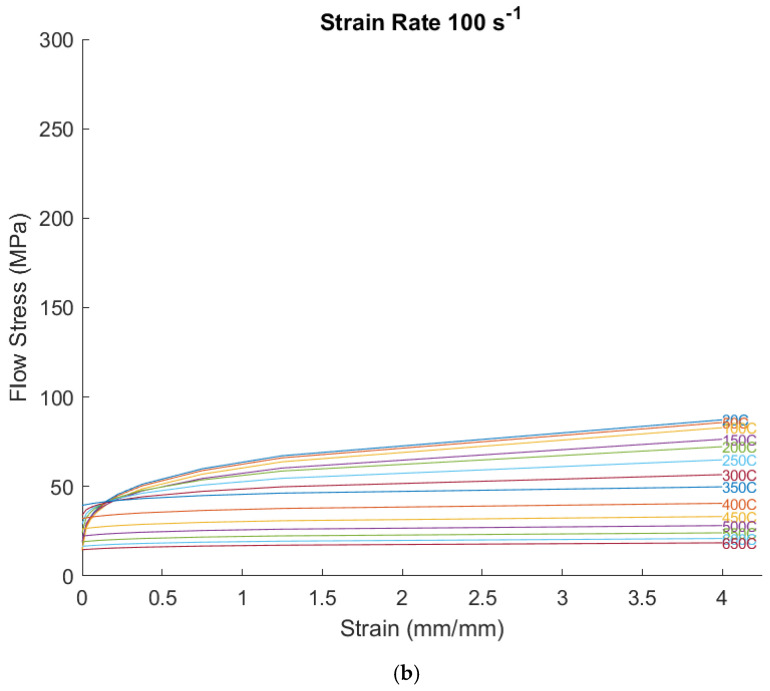
Flow stresses at a strain rate of 100 s^−1^ using (**a**) the standard AA2219 data from hot compression/hot torsion and (**b**) the modified AA2219 high-pressure shear test results, reflecting the reduced flow stresses measured near a spinning FSW tool.

The standard AA 2219-T76 flow stress data available from JMatPro were compared to the experimental measurements from a newly developed high-pressure shear test approach at different strain rates and temperatures performed in a prior work [9]. The high-pressure shear test better replicates the high shear conditions that occur next to a rapidly rotating friction stir welding tool and results in lower flow stresses than those generated by hot compression or hot torsion testing for the high strain rates and temperatures that occur during FSW. Metallurgically, as discussed in the prior work, the continuous dynamic recrystallization that occurs during high shear deformation at elevated temperatures, results in a sort of high strain rate grain boundary sliding, and therefore lower flow stresses, compared to hot compression and hot torsion. As such, a simple linearized factor was used to correlate the existing JMatPro flow stress curves with the high-pressure shear experimental data from the new test method. Two data sets were compared at a strain rate of 100 s^−1^, assuming that the flow stresses reach saturation rapidly at these strains and strain rates. The comparison of before and after is shown in Figure 13. The curves are close together in Figure 13b, so it is not easy to see each individual curve, but the y-axis limit of 300 MPa was retained, so it shows the relative differences between them (Figure 13a,b).

**Figure 14:**
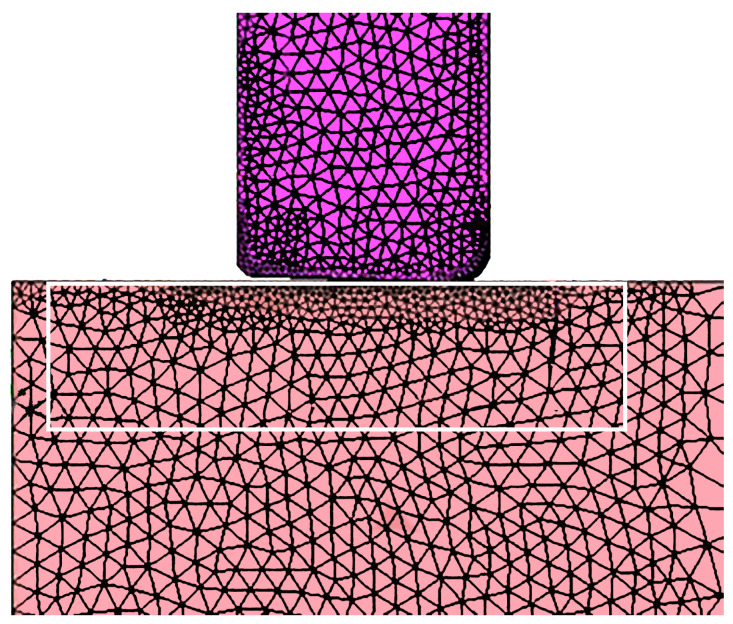
The area of the AA2219 plate where more accurate local flow stresses were integrated into the model. Outside the white box, standard flow stresses from hot compression/hot torsion were employed (applies to the plate portion only; the tool had its own properties for H13 steel). Note that the axis of symmetry in this case is at the left edge of the plate mesh.

The modified flow stresses were then implemented into the model. Since the majority of the plate does not undergo significant plastic deformation and high strain rates, the modified material properties only needed to be assigned to the portion of the plate mesh just under the tool, where a shear layer is observed experimentally (see Figure 14). Outside this zone, the standard AA2219 flow stresses were applied. The specified rheology area was made to be slightly larger than the flow zone to cover all potential large deformations during processing.

**Figure 15:**
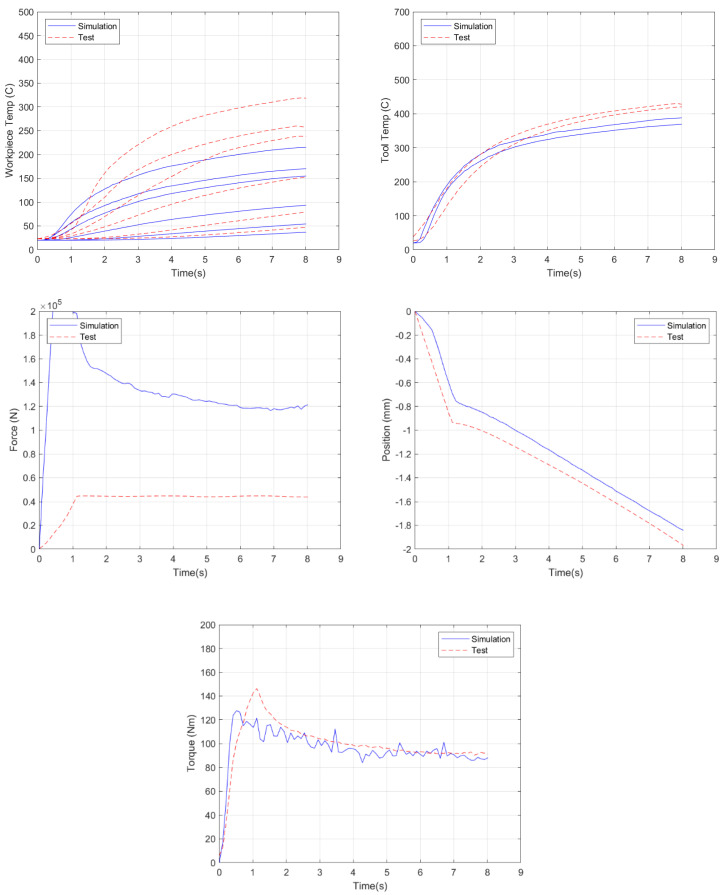
Simulation results for the standard 2219 rheology and viscoplastic friction parameters (α = 0.20, *p* = 0.15).

Given the results of the prior section, where the viscoplastic friction law was seen to provide more accurate simulation predictions, a more rigorous model validation was carried out using this law. As a baseline comparison, the model was run with the standard JMatPro AA 2219 material properties, while, at the tool–plate interface, the viscoplastic friction law default values of 0.20 and 0.15 for *α* and *p*, respectively, were employed, as shown in Figure 15. This resulted in an overall error of 39.7%. While the torque and tool temperature results had less than 10% error, the other results were not as accurate. The plate temperatures were underpredicted, with an average error of 24%, while the forces were overpredicted, with an average error of 282%.

**Figure 16:**
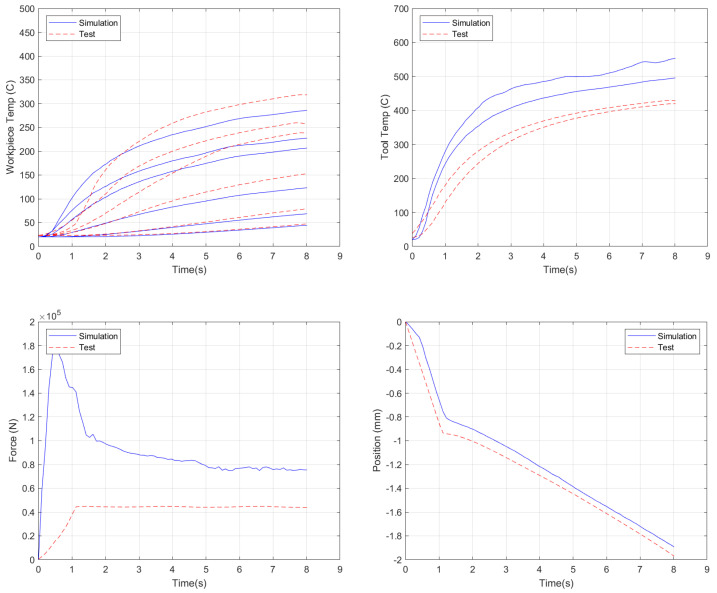
Simulation results using the standard AA2219 flow stress data and viscoplastic friction parameters of α = 0.30 and *p* = 0.20.

Next, the same model was run, but the friction parameters were changed to the best parameter values from the characterization study: *α* = 0.30 and *p* = 0.20 (*α* = 0.40 and *p* = 0.15 provide very similar results and are now shown); see Figure 16. It should be noted that these simulations do not include torque due to convergence issues, where, initially, insufficient nodes were in contact between the tool and plate. In order to keep the error comparisons comparable, the torque results were excluded from the error calculations here. For comparison, the baseline simulation run had an error of 42.7% without torque included.

These parameter sets improved upon the baseline simulation results, except that the force improved to only 200% of the experimental values versus the nearly 300% present in the baseline case. Significant improvements were made to the plate temperatures, while the tool temperatures were still overpredicted. These two runs generated an overall error reduction of approximately 20%, with overall simulation errors of 33.5%.

**Figure 17:**
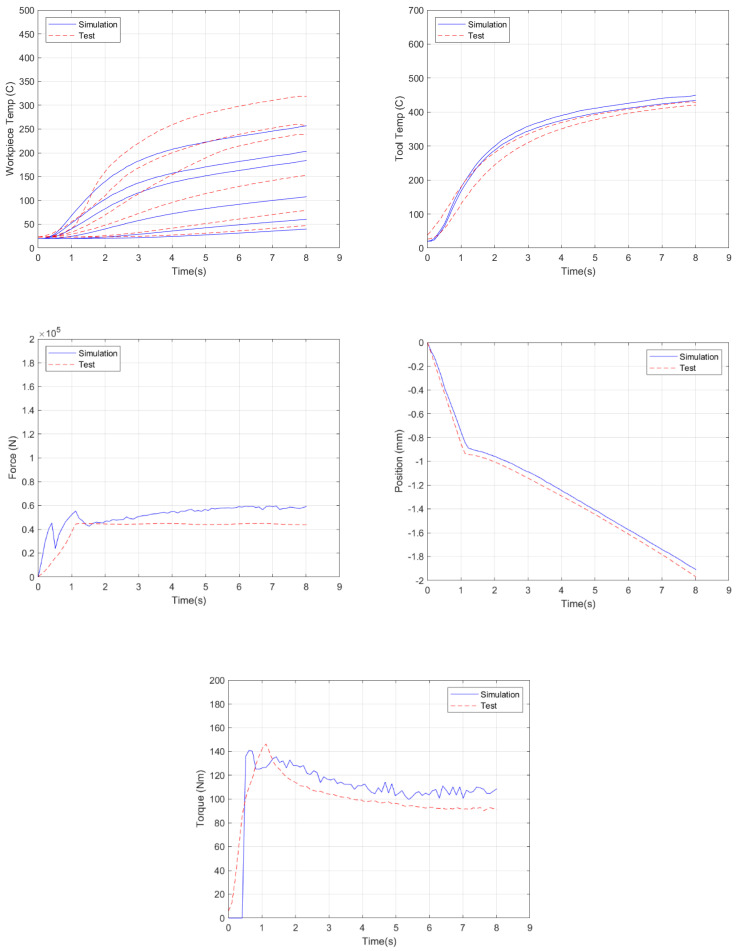
Simulation results using the modified AA 2219 flow stresses and viscoplastic friction parameters α = 0.30 and *p* = 0.20.

To further improve the model predictions, the flow stresses measured by the high-pressure shear method were implemented in the portion of the plate mesh (Figure 13) under the tool. Convergence issues related to the torque calculation were also resolved. As such, the overall error was reduced to 18.7% for the case shown in Figure 17. The majority of the error reduction was in the force results, with a reduction of 78.5% from the baseline, owing to the improved flow stress data used in the plate mesh in the area under the tool. The improved flow stresses also improved the accuracy of the tool and plate temperatures, especially in the first 2 s of the simulation, by reducing the rate at which these temperatures increased initially.

## 5. Conclusions

The modeling of friction stir welding (FSW) is challenging, particularly in two aspects: the flow stresses needed to represent the material behavior across a broad range of strain rates and temperatures are difficult to measure with traditional methods, and the friction laws that are used to model heat generation have not been adapted to the case of high-velocity sliding of an FSW tool on a plate. As such, the current work employed a recently developed method to measure flow stresses in AA 2219-T67 at the high strain rates typical of FSW. These data were used in the development of a finite element simulation of FSW to study the effect of the new flow stress data on the temperature, torque, and load predictions compared to material models calibrated with hot compression or hot torsion data. It was found that load predictions, for example, were significantly better with the new material law, reducing the error with respect to the experimental measurements by approximately 79% over the duration of the plunge.

In addition, the characterization of the Tresca-limited Coulomb and viscoplastic friction laws led to a better understanding of how the key parameters in each law affect heat generation and the resulting temperature profiles in both tool and plate. This characterization led to a down selection of the viscoplastic law, with optimized parameters for *α* and *p* of 0.30 and 0.20, respectively, for the modeling of an FSW plunge in AA 2219-T76, as it produced the lowest error when considering both the transient and steady-state phases of welding. Thus, the primary conclusions from this work are as follows:

- The viscoplastic friction law was found to be the most appropriate for modeling FSW, because it accurately captures both the transient and steady-state phases of an FSW plunge experiment, owing to its rate sensitivity to local shearing of the plate material.
- The properly tuned friction law parameters provided a significant improvement in simulation accuracy compared to the benchmark model using a set of typical parameters. Accurate modeling of friction in FSW is an area where future research could improve the predictive capability of modeling, as the current laws have been adapted from processes like metal forming that do not have the intense shear deformation that is observed in FSW.
- It was shown that using flow stresses measured at the high strain rates and temperatures characteristic of friction stir welding, combined with optimized friction law parameters, provided a reduction in simulation error from 53.8% to 18.7% (65% reduction) when comparing predictions obtained with the baseline friction parameters and flow stress data generated by hot compression or hot torsion testing. Overall error was calculated as an equally weighted comparison of temperatures, torques, and forces with experimentally measured values.

## Figures and Tables

**Table 1:** Summary of the friction laws used in this study.

Friction Law	Description	Equation
Tresca	Frictional resistance limited to the yield stress of the material in pure shear	(1)
Tresca-limited Coulomb	Coulomb friction that saturates to Tresca friction	(3)
Viscoplastic (Norton)	Shear stress as a function of relative sliding velocity and local flow stress	(4)

**Table 2:** Heat transfer coefficient at each contact interface of the model.

Model Interface	$\mathrm{Thermal}\;\mathrm{Exchange}\;\mathrm{Coefficient}\;\left[\frac{W}{m^{2}\cdot K}\right]$
Tool—Tool Holder	20,000
Tool—Plate	20,000
Plate—Backing Plate	2000
Backing Plate—Backing Plate Holder	Adiabatic

**Table 3:** Results used to calculate the error of a simulation result.

Simulation Results Being Compared to the Experiment	Number of Variables
Plate Temperatures	6
Tool Temperatures	3
Force on Tool	1
Torque on Tool	1
Position of Tool	1

**Table 4:** Simulation errors using the viscoplastic friction law (lowest errors and best parameters in bold).

α	*p*	Error (%)
0.30	0.15	77.3
**0.30**	**0.20**	**62.3**
0.30	0.25	68.3
0.30	0.30	84.4
**0.40**	**0.15**	**53.9**
0.40	0.20	66.3
0.40	0.25	82.4
0.40	0.30	86.0

## Data Availability

The original contributions presented in this study are included in the article. Further inquiries can be directed to the corresponding author.
